# Atypical Outcomes of Nasal and Lip Appearance After Unilateral Cleft Lip Repair: Judgment by Professionals, Patients, and Laypeople

**DOI:** 10.1177/1055665620982801

**Published:** 2021-01-19

**Authors:** Robin A. Tan, Frans J. Mulder, Roderic M. F. Schwirtz, David G. M. Mosmuller, Henrica C. W. De Vet, J. Peter W. Don Griot

**Affiliations:** 1 Department of Plastic, Reconstructive and Hand Surgery, 1209 Amsterdam UMC, location VUmc, Amsterdam, the Netherlands; 2 Department of Epidemiology and Data Science and the Amsterdam Public Health, Research Institute, 1209 Amsterdam UMC, Location VUmc, Amsterdam, the Netherlands

**Keywords:** cleft lip and palate, nasolabial appearance, photographic evaluation, assessment, survey

## Abstract

**Objective:**

To gain more insight into the assessment of “atypical” nasal and lip appearance outcomes compared to “typical” appearance outcomes after unilateral cleft lip and palate (UCLP) repair, when judged by professionals, patients with repaired UCLP, and laypeople.

**Design:**

An online survey containing 3 series of photographs with various degrees of “typical” and “atypical” nasal and lip appearance outcomes after UCLP repair was sent to 30 professionals, 30 patients with repaired UCLP, and 50 laypeople in 2 countries. Participants were instructed to rank the photographs from excellent to poor based on overall appearance. Mean rank positions of photographs were analyzed and differences in mean rank score between “typical” and “atypical” results were assessed using a T-test. Agreement of ranking between the 3 groups was assessed with an analysis of variance analysis.

**Setting:**

Amsterdam UMC, location VUmc, Netherlands and Boston Children’s Hospital, Boston, USA.

**Patients:**

Photographs of 6- to 18-year-old patients with repaired UCLP.

**Results:**

“Atypical” appearance outcomes were ranked significantly less favorably (small nostril: *P* = 0.00; low vermillion border: *P* = 0.02; whistling deformity: *P* = 0.00) compared to “typical” outcomes. Difference between professionals, patients and laypeople in rank positioning the photographs was not statistically significant (*P* = 0.89).

**Conclusions:**

Noses with a smaller nostril and lips containing a whistling deformity were perceived as poorer outcome compared to the “typical” results. Professionals, patients, and laypeople are in agreement when assessing these outcomes.

## Introduction

**Figure 1.**
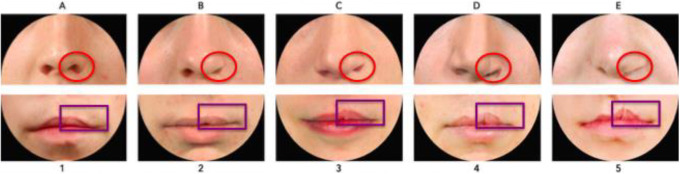
The Cleft Aesthetic Rating Scale (CARS). The CARS was designed to grade five degrees of  “typical” nasal and lip outcomes after left-sided UCLP repair. Raters assess symmetry between left and right side, concerning the degree of alar flaring (red circles) and upper lip vermillion border height (purple rectangles). The nose/lip receive separate scores from A-E/1-5 (excellent to poor).

In patients with repaired unilateral cleft lip and palate (UCLP), the nasolabial appearance seems to substantially influence quality of life and patient satisfaction besides other treatment-related factors, such as facial and psychological functioning (Mani et al., 2013; Klassen et al., 2018; Wong Riff et al., 2018). To evaluate and compare such appearance outcomes, an easy-to-use and generally accepted evaluation method is needed (Al-Omari et al., 2005; Sharma et al., 2012; Mosmuller et al., 2013). The Cleft Aesthetic Rating Scale (CARS) (Mosmuller et al., 2017; Mulder et al., 2018) was developed and validated to evaluate postoperative appearance of the nose and lip in 6-and 18-year-old patients with repaired UCLP. The CARS was designed to grade 5 degrees of severity of “typical” appearance outcomes (Figure 1) of the nose and lip separately, using a photographic reference scale. A “typical” nasal outcome refers to a wider nostril on the repaired cleft-side (alar flaring) and ranges from symmetrical (A) to very wide (E). The degree of flaring often worsens over time and may vary depending on several factors such as surgical scarring, changes related to growth, and the surgical technique used (Choi et al., 2012). A “typical” lip outcome refers to the upper lip, and ranges from a symmetrical Cupid’s bow to a highly retracted vermillion border, also on the repaired cleft side. The latter development, caused by scar contraction, is commonly seen in patients with repaired UCLP (Farkas et al., 1993; Ayoub et al., 2011; Heller et al., 2011; Al-Rudainy et al., 2018). Outcomes such as a narrower nostril, a depressed vermillion border, or a “whistling deformity” (characterized by tissue loss in the medial tubercle of the lips) on the repaired cleft side, on the other hand, are seen less often (Bozkurt et al., 2010) and are therefore labeled as “atypical” outcomes. These 3 “atypical” outcomes were not included in the CARS as reference photographs, so raters did not know how to grade these results. As a result there is no answer to the question whether “atypical” outcomes are assessed as poor, fair, or good.

Literature on how these “typical” and “atypical” outcomes are judged in relation to each other is lacking. We therefore conducted an online survey in the current study.

The objectives of this study were: (1) To assess how various degrees of both “typical” and “atypical” appearance outcomes are related to each other when being assessed, and more specifically whether a “typical” or an “atypical” result is perceived as most favorable. (2) To determine whether health care professionals, patients with repaired UCLP, and laypeople are in agreement when judging the various appearance outcomes.

## Methods

**Figure 2.**
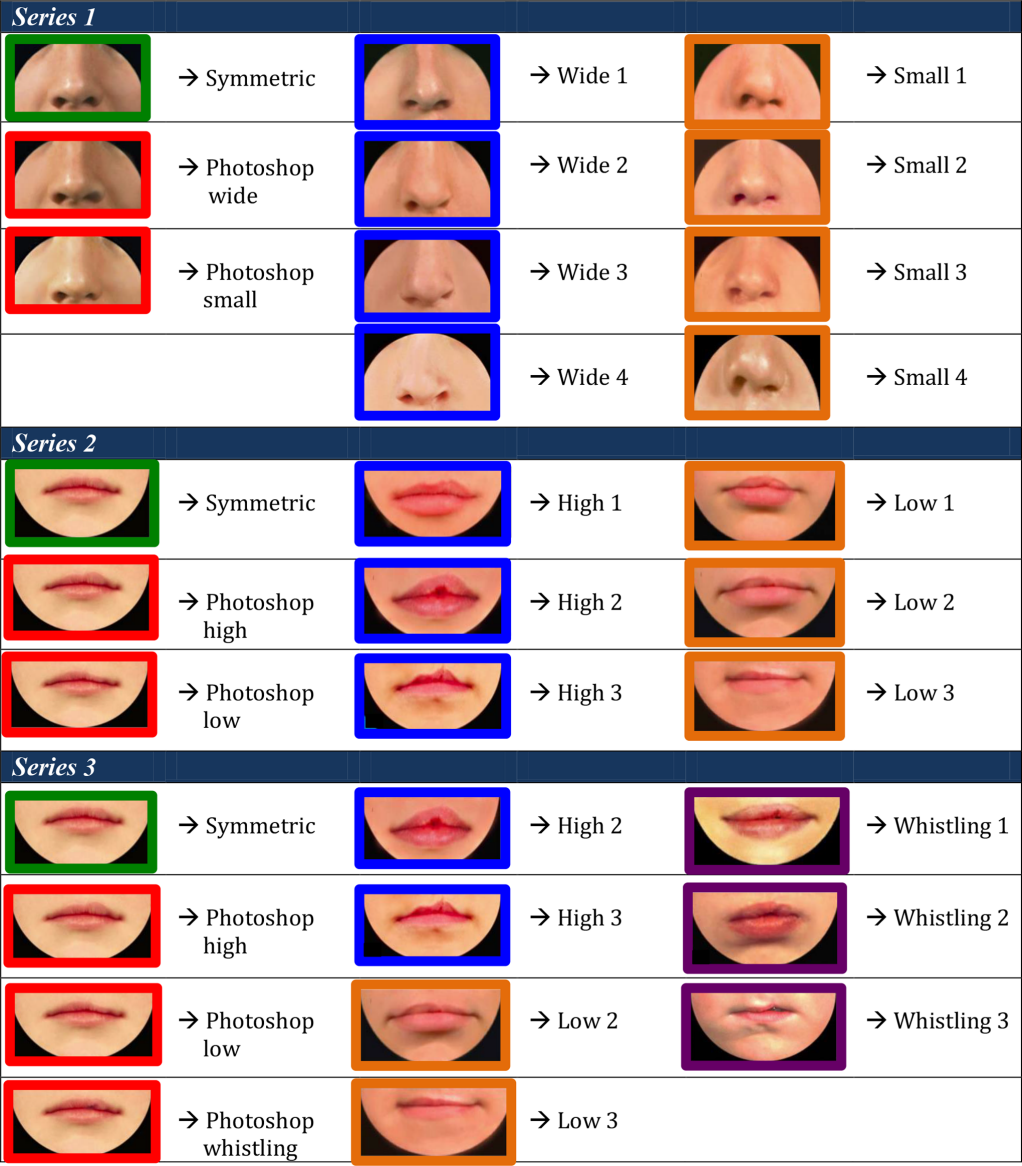
Photographs included in each series. All repaired clefts are presented as left-sided. Green = Most symmetric nose/lip (100% inter-rater score of A/1 from the three raters), Red = Edited versions of most symmetric nose/lip. Blue = Typical outcomes, i.e. noses/lips with increasingly wider nostrils/higher vermillion borders. Higher score = increasing asymmetryOrange = Atypical outcomes,  i.e. noses/lips with increasingly narrow nostrils/lowervermillion borders. Increasing numbers = increasing asymmetryPurple = Atypical outcomes, i.e. lips with whistling deformity. Higher score = increasing asymmetry.

Cropped frontal photographs of noses and lips of 18-year-old patients who underwent UCLP repair were collected and graded by 3 raters (2 cleft surgeons and 1 cleft orthodontist) using the CARS to grade “typical” nasal and lip outcomes (Mulder et al., 2018). All photographic material was obtained from the Amsterdam UMC affiliated Academic Center for Dentistry Amsterdam database, where patient photographs are stored. Skin tone and any unevenness was corrected on the included photographs using Photoshop_._ With these photographs, an online survey was created to compare “typical” and “atypical” appearance outcomes by rank ordering 3 photographic series (Figure 2).

Series 1 consisted of 11 photographs and was created to assess how a nose with a smaller nostril (“atypical” outcome) is ranked compared to a nose with a wider nostril (“typical” outcome). A photograph with an excellent symmetrical outcome (green) was selected, which all 3 raters in the study of Mulder et al. (2018) scored with an “A.” Next, this photograph was digitally edited using Photoshop (Adobe Systems Inc) to represent a “typical” and an “atypical” outcome (red). To complete the set, 4 different photographs of a patient with a wide nostril (blue) and 4 with a small nostril (orange) were added, to represent a range of severity of “typical” and “atypical” outcomes, respectively (Figure 2). Preferably, the “typical” photographs were selected with a 100% interrater CARS score.

Series 2 consisted of 9 photographs and was created to assess how a lip with a low vermillion border (“atypical” outcome) is ranked compared to a lip with a high vermillion border (“typical” outcome). Similar to Series 1, a photograph with a symmetrical lip outcome was selected (green) and digitally edited to create a “typical” and an “atypical” lip (red). Added were 3 photographs with a “typical” high (blue) and “atypical” low (orange) vermillion border, representing a wide range of lip outcomes. Eleven photographs were included in series 3, consisting of the 2 most severe “typical” and “atypical” lip outcomes (from the range of outcomes in series 2), as well as a range of photos of patients with whistling deformities (purple), to compare this deformity to a high or low vermillion border (Figure 2).

Using these 3 series, a survey was created on the online platform Qualtrics (Qualtrics presenting the photographs in random order. Participants were instructed to rank order the 11, 9, and 11 photographs of the first, second, and third series, respectively, by dragging the photographs toward the position of preference with the computer mouse in order of severity, from an excellent (top) to a very poor (bottom) outcome based on overall appearance. To aid the rank-ordering task, raters were instructed to place the 2 best outcomes in the first and second positions and the 2 worst outcomes in the second-to-last and last positions.

The aim was to obtain completed online surveys from 30 professionals involved in cleft lip and palate care, 30 patients with repaired UCLP, and 50 laypeople with no medical background. All participants were at least 18 years old. Email addresses of participants were obtained and an instruction letter was sent which contained an anonymous link providing access to the survey. The email inviting participants to complete the online survey was sent on behalf of Amsterdam UMC, location VUmc, Amsterdam, Netherlands (NL), and Boston Children's Hospital, Boston, MA, USA. Informed consent was obtained within the online module. Institutional review board approval was granted from the Medical Ethical Committee of both institutions.

### Statistical Analysis

Microsoft Excel (Microsoft Corp) and the statistical program SPSS (IBM Corp) version 24.0 were used to analyze the data. The mean rank scores and standard deviations (SDs) were calculated for each photograph for the professionals, the patients, and the laypeople. Next, differences in mean rank positioning of the photographs between those 3 groups were determined using an analysis of variance. The edited photographs (red) were used as the gold standard to assess whether the typical or the atypical outcome was regarded as more favorable. These differences in rank order position of the edited photographs were analyzed in all 3 series using an independent sample T-test. Sample size was determined based on a balance between burden of participating for professionals and patients and the guarantee of robust, reliable data to enable drawing conclusions.

## Results

The survey was kept online from September 2016 to June 2018 until the desired sample size was achieved. A total of 30 professionals (NL: 24; USA: 6), that is, 20 cleft-surgeons and 10 cleft-care involved orthodontists, 30 patients with repaired UCLP (NL: 11; USA: 19), and 50 laypeople (NL: 50; USA: 0) completed the survey. Differences in mean rank positions did not differ statistically significant between the professionals, patients, and laypeople within each of the 3 series (series 1: *P* = 0.94; series 2: *P* = 0.87; series 3: *P* = 0.85). A total (column 1) reflecting the mean rank positions of the sum of the 3 groups is therefore presented in Tables 1, 2, and 3.

**Table 1.** Comparison of mean rank positions of nasal photographs of patients with a “typical” wider nostril and an “atypical” smaller nostril in series 1.

Total, (N = 110) Position. mean position (SD)	Photograph	Description	Professionals (N = 30) Position. mean position (SD)	Patients (N = 30) Position. mean position (SD)	Lay people (N = 50) position. mean position (SD)
1. 1.86 (0.90)	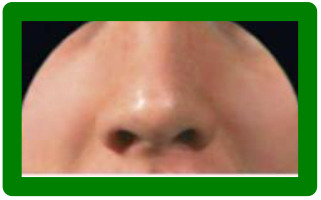	Symmetric	1. 1.63 (0.56)	1. 1.73 (0.98)	2. 2.08 (0.99)
2. 2.01 (0.98)	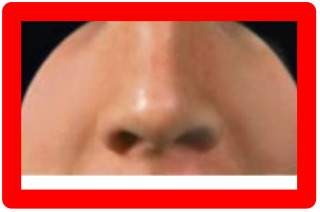	Photoshop wide	2. 1.93 (1.05)	2. 2.13 (0.90)	1. 1.98 (1.00)
3. 2.45 (1.11)	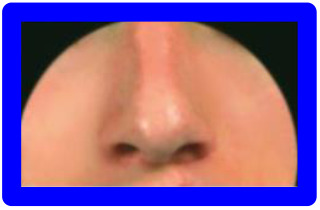	Wide 1	3. 2.70 (0.92)	3. 2.33 (0.76)	3. 2.38 (1.35)
4. 4.41 (1.24)	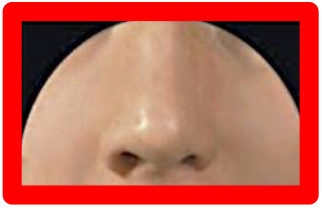	Photoshop small	4. 4.23 (1.14)	4. 4.30 (1.29)	4. 4.58 (1.26)
5. 6.30 (1.76)	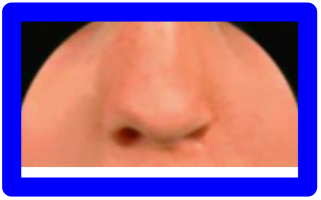	Wide 2	5. 6.13 (1.57)	5. 6.77 (1.77)	5. 6.12 (1.82)
6. 7.16 (1.82)	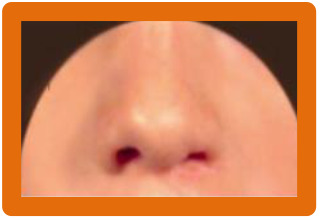	Small 2	6. 7.00 (1.97)	6. 7.13 (1.89)	7. 7.28 (1.71)
7. 7.44 (1.84)	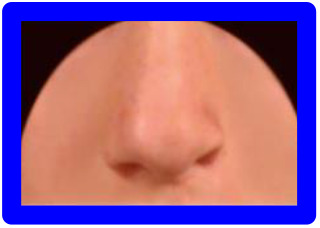	Wide 3	9. 8.53 (1.48)	7. 7.20 (1.69)	6. 6.92 (1.87)
8. 7.44 (1.84)	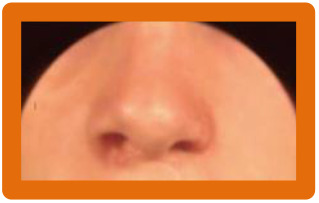	Small 3	7. 7.10 (1.65)	8. 7.30 (1.97)	8. 7.72 (1.85)
9. 7.86 (1.66)	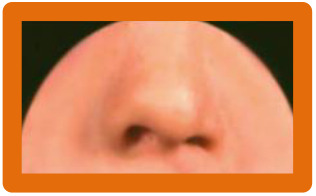	Small 1	8. 7.50 (1.55)	9. 7.73 (1.68)	10. 8.16 (1.68)
10. 8.36 (2.11)	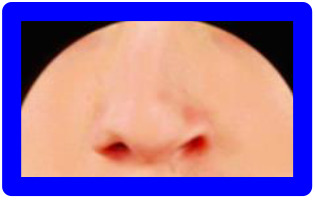	Wide 4	10. 8.80 (2.17)	10. 8.73 (1.91)	9. 7.88 (2.13)
11. 10.70 (0.70)	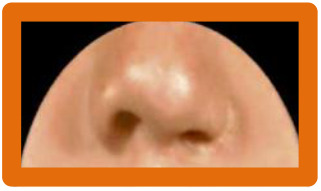	Small 4	11. 10.43 (0.94)	11. 10.63 (0.69)	11. 10.90 (0.46)

Abbreviation: SD, standard deviation.

**Table 2.** Comparison of mean rank positions of lip photographs of patients with a “typical” high vermillion border and an “atypical” low vermillion border in series 2.

Total (N = 110) position. **mean position (SD)**	Photograph	Description	Professionals (N = 30) position. **mean position (SD)**	Patients (N = 30) position. **mean position (SD)**	Lay people (N = 50) position. ***mean position* (SD)**
1. 1.59 (1.30)	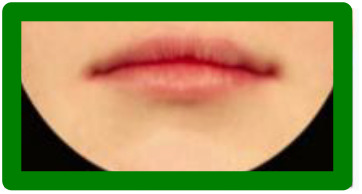	Symmetric	1. 1.30 (1.21)	1. 1.53 (1.07)	1. 1.80 (1.46)
2. 3.03 (1.47)	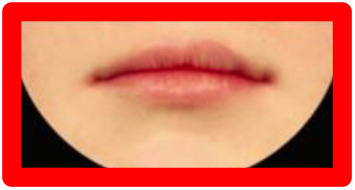	Photoshop high	2. 2,50 (0.94)	2. 3,33 (1.42)	2. 3.16 (1.68)
3. 3.64 (1.57)	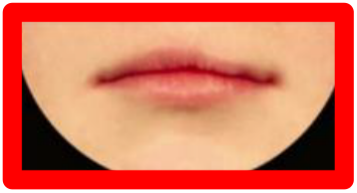	Photoshop low	3. 3,10 (0.96)	3. 4,03 (1.83)	3. 3.72 (1.63)
4. 4.72 (1.93)	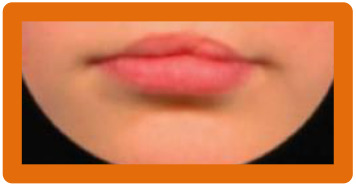	Low 1	5. 5,10 (1.45)	5. 4,67 (2.28)	4. 4.52 (1.95)
5. 4.82 (1.86)	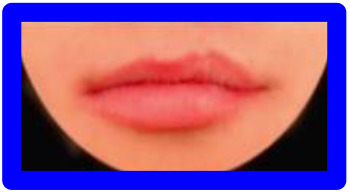	High 1	4. 4.97 (2.01)	4. 4.57 (1.79)	6. 4.88 (1.83)
6. 5.35 (1.74)	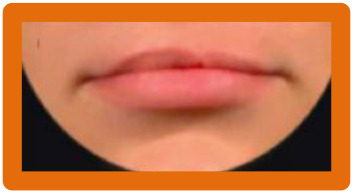	Low 2	7. 6,13 (1.31)	7. 5,43 (1.94)	5. 4.84 (1.68)
7. 5.60 (1.81)	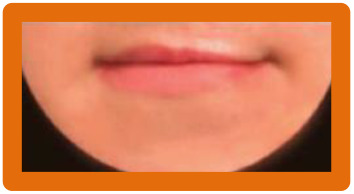	Low 3	6. 5.80 (1.54)	6. 5.30 (1.93)	7. 5.66 (1.89)
8. 7.52 (1.25)	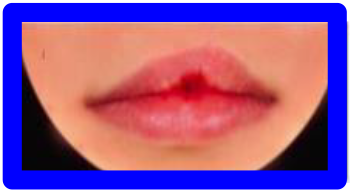	High 2	8. 7,50 (0.94)	8. 7,40 (1.43)	8. 7.60 (1.31)
9. 8.75 (0.68)	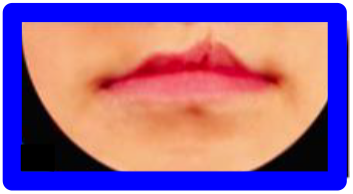	High 3	9. 8,67 (0.96)	9. 8,73 (0.58)	9. 8.82 (0.52)

Abbreviation: SD, standard deviation.

**Table 3.** Comparison of mean rank position of lip photographs from patients with a “typical” high vermillion border, an “atypical” low vermillion border, or a whistling deformity in series.

Total (N = 110) position. **mean position (SD)**	Photograph	Description	Professionals (N = 30) position. **mean position (SD)**	Patients (N = 30) Position. **mean position (SD)**	Lay people (N = 50) position. **mean position (SD)**
1. 1.97 (1.58)	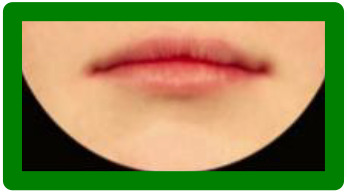	Symmetric	1. 1.17 (0.53)	1. 2.27 (1.80)	1. 2.28 (1.71)
2. 2.90 (1.37)	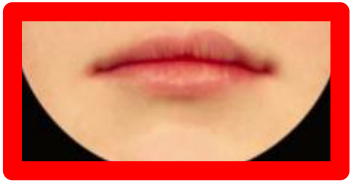	Photoshop high	2. 2.70 (0.99)	2. 3.13 (1.48)	2. 2.88 (1.49)
3. 3.76 (1.73)	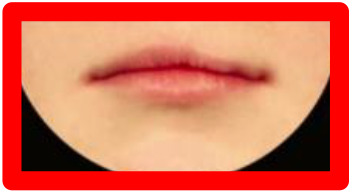	Photoshop low	3. 3.60 (1.38)	3. 4.00 (2.18)	3. 3.72 (1.63)
4. 4.31 (1.93)	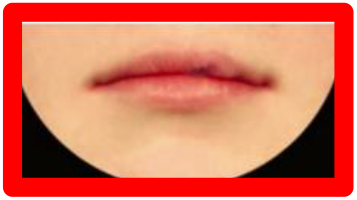	Photoshop whistling	4. 4.13 (2.15)	4. 4.27 (1.76)	4. 4.44 (1.92)
5. 4.97 (2.34)	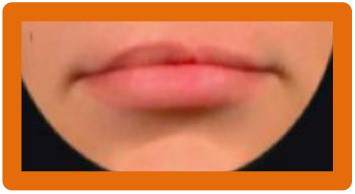	Low 1	5. 5.80 (2.11)	5. 4.77 (2.60)	5. 4.60 (2.24)
6. 5.29 (2.05)	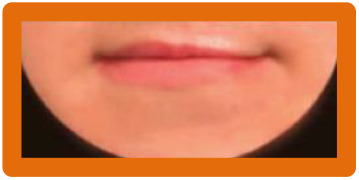	Low 2	7. 5.97 (1.79)	6. 5.17 (2.17)	6. 4.96 (2.07)
7. 6.91 (2.13)	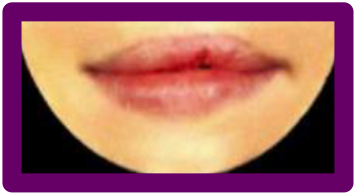	Whistling 1	6. 5.83 (1.40)	7. 6.57 (2.34)	8. 7.78 (2.01)
8. 7.49 (1.94)	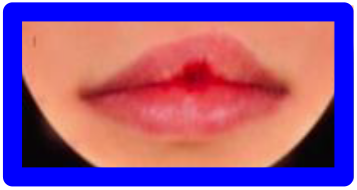	High 1	8. 8.10 (1.35)	8. 7.37 (2.09)	7. 7.20 (2.10)
9. 8.79 (1.54)	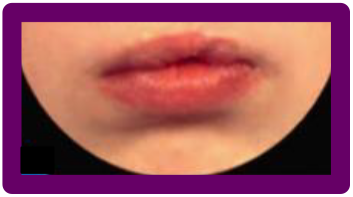	Whistling 2	9. 8.70 (1.39)	9. 8.67 (1.65)	9. 8.92 (1.58)
10. 9.47 (1.61)	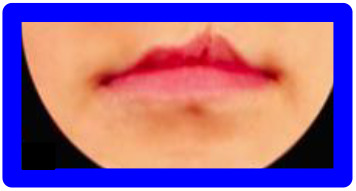	High 2	10. 9.80 (1.03)	10. 9.70 (1.88)	10. 9.14 (1.68)
11. 10.08 (1.27)	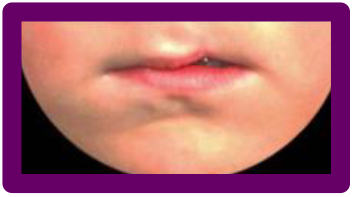	Whistling 3	11. 10.23 (0.97)	11. 9.93 (1.11)	11. 10.08 (1.51)

Abbreviation: SD, standard deviation.

Regarding the nasal outcomes (Table 1), the most symmetrical nose (green) was ranked as having the best overall appearance in the total group (column 1), with a mean rank position of 1.86 (SD: 0.90). When comparing the 2 edited versions (red), the appearance of the “typical” wider nostril was ranked as significantly better than the “atypical” smaller nostril (mean rank position of 2.01 [SD: 0.98] vs 4.41 [SD: 1.24], respectively; *P* < 0.001). Within the professionals group (column 4), photograph “Wide 3” was perceived as a worse outcome (mean rank 8.53) than photographs “Small 1-3” (mean rank position between 7.00 and 7.50). By contrast, the lay people (column 6) ranked photograph “Wide 3” (mean rank position 6.92) higher than photographs “Small 1-3” (mean rank position 7.28 and 8.16). Overall (column 1), the 4 noses with a wider nostril (blue) received higher mean rank scores than those with a smaller nostril (orange). Yet, the most asymmetrical wider (“wide 4”) as well as the smaller (“small 4”) nostrils were ranked as the worst appearance outcomes (mean rank position 8.36 [SD: 2.11] and 10.7 [SD: 0.70], respectively).

The mean rank position calculated for the 4 noses with smaller nostrils (column 1) was 8.29 (SD: 1.51). This mean score mostly resembled the score 8.36 (SD: 2.11) of the “typical wide 4” nose that received a “D” score according to the CARS in the study of Mulder et al. (2018).

Concerning the upper lip, looking at the total group (Table 2, column 1), the most symmetrical lip (green) was placed in the highest mean rank position of 1.59 (SD: 1.30). The edited lip (red) with a higher upper lip vermillion border was ranked significantly (*P* = 0.02) better than the edited lip (red) with the lower vermillion border. However, looking at the ranking of the other photographs of a “typical” high (blue) and “atypical” low (orange) vermillion border, patients with a low vermillion border were perceived as better outcomes.

The mean rank position calculated for the 4 lips with a lower vermillion border (column 1) was 5.22 (1.83). This score mostly resembled lip “high 1” (4.82 SD: 1.86) which received a CARS score of “3.”

When comparing these typical and atypical outcomes to patients with a whistling deformity (Table 3, column 1), whistling deformity was rated as the poorest outcome. It had the lowest mean rank position of the (subtly) edited images, ranking lower than the edited high vermillion border (*P* < 0.001) and low vermillion border (*P* = 0.03) in the total group. Additionally, when looking only at the most asymmetrical photographs (Low 2, High 2, and Whistling 3), the whistling deformity was ranked as poorest outcome as well. Moreover, the patient photographs with a whistling deformity were all positioned in the bottom half of the ranking. The mean score calculated for the 3 whistling deformity lips (column 1) was 8.59 (1.69) and this score resembled lip “high 2” (9.47 SD: 1.61) in series 3, which received a CARS score of “4.”

## Discussion

The purpose of this study was to gain more insight into the assessment of various “typical” and “atypical” outcomes of the nose and lip after UCLP repair. The 2 objectives outlined in the introduction section (1. perception of most favorable outcome; 2. agreement of different rater groups on assessments) are addressed below.

As expected, the larger the asymmetry between the repaired cleft side and the non-cleft side, the worse the ranking, regardless of whether it was a “typical” or an “atypical” outcome. Generally, a “typical” wider nostril is perceived as more favorable than an “atypical” smaller nostril. For the lip, the (subtly) edited higher vermillion border was ranked as better than an “atypical” low vermillion border, although in case of a larger asymmetry, photographs with a low vermillion border were ranked better. Lips with a whistling deformity are the least favorable outcome when compared to a lip with a higher or a lower vermillion border on the repaired cleft side. We found no significant differences in ranking of the photographs by health care professionals, patients with repaired UCLP and laypeople.

Although no literature is available on how these various outcomes are related to each other when evaluated on appearance, earlier studies on the incidence of secondary revisions could provide an indication for preferences in appearance outcomes among patients and professionals. One can expect that a higher incidence of a particular secondary surgical procedure will involve patient dissatisfaction regarding their appearance. Most of the secondary revisions are performed to address asymmetries (ie, appearance) in the nasal and lip area (Mackay et al., 1999; Rothermel et al., 2020). Nevertheless, reasons to perform surgical UCLP repair for an “atypical” small nostril and an “atypical” low vermillion border have not been described explicitly in literature. A review by Sitzman et al. (2016) shows that the majority of lip revision surgery is performed to correct deformities of the vermillion border, without mentioning, however, whether these were “typical” or “atypical” deformities. Scheller et al. (2019) reported that a whistling deformity is the most corrected secondary deformity concerning the lip, and Henkel et al. (1998) reported that out of 126 patients with repaired UCLP, 17 (13%) patients received corrective surgery due to inequality in lip segments and for 36 (29%) patients the reason for revision was a whistling deformity. Also, Mulliken & Martinez (1999) assessed secondary revision rates in 105 patients with repaired UCLP. Of these patients, 27% (n = 28) were treated to correct the free mucosal margin (ie, whistling deformity). The relatively high revision rates with regard to a whistling deformity indicate that this type of deformity is commonly denoted as a relatively poor outcome that demands corrective surgery. This is in line with the results of our study.

No differences in assessment were found between the professionals, patients, and laypersons. However, we focused on severity ranking, which means there can still be differences between these groups in terms of absolute scores. The discrepancy in nasolabial appearance assessment between professionals, patients, and laypeople has been reported before, although most studies did not include a patient group. In the review by Zhu et al. (2016), 11 studies evaluated assessment of full-face patient photographs by professionals and laypeople. Their conclusion was that it is still not clear whether professionals and laypeople are in agreement, because 3 studies found that laypeople were more critical than professionals, 3 found there was no significant difference between laypeople and professionals, and 5 reported that professionals were more critical than laypeople. More recently, Mulder et al. (2018) concluded that there were no differences between professionals and laypeople in the assessment of nasolabial appearance on cropped photographs using the CARS. Adetayo et al. (2018) found that there were no significant differences when professionals and laypeople graded the lip, but for grading the nose laypeople reported significantly poorer scores, using direct panel assessment. Alhayek et al. (2019) reported that professionals rated facial appearance of patients with repaired clefts significantly lower than laypeople using a visual analogue scale, but professionals had a higher perceived need for further treatment. As long as there is a mismatch in appearance assessment after UCLP repair, regardless of outcome evaluation tools, a strong emphasis should remain on clear communication between the physician and patient regarding their expectations, perception, and satisfaction regarding surgical results (Mulder et al. 2018).

### Strengths and Limitations

The use of an online platform to distribute a survey or a questionnaire in which participants were asked to rank order or to grade photographs is a recently popularized method (Tse et al., 2016; Van Veldhuisen et al., 2017). Such a method makes it possible to reach numerous participants globally. Moreover, rank ordering photographs seemed to be an effective and reliable method for evaluating patients with cleft lip and palate (Fisher et al., 2008; Mercan et al., 2018). In this study, the use of a rank ordering method was valuable, since participants were able to easily distinguish between the different outcomes. In addition, the rank ordering task was simplified because the participants could drag the photographs into the preferred position using the computer mouse. On the other hand, as rank ordering forces a participant to place a photograph after or before another, there is no possibility to rank photographs as identical in terms of outcome.

In order to assess if a “typical” or an “atypical” outcome is perceived as most favorable, edited versions (Photoshop) of the most symmetrical nose and lip photographs were incorporated in the survey. In this way, confounding factors such as skin color and variety in anatomical structures were eliminated, leaving only the “typical” or “atypical” aspect visible. This procedure may have increased the effectiveness and reliability of the rank ordering task.

A few limitations need to be addressed. First, the survey was distributed in 2 countries (NL and USA), and potential geographic bias may therefore have occurred. As the majority of the participants were recruited in The Netherlands, the results in this study may only represent appearance preferences of this specific region. since the ratio of NL and USA participants was not proportional, further investigation of possible differences between both countries was decided against.

Second, most participants completed the survey in their work or home setting. Potential rushing or submitting the survey as incomplete is a potential shortcoming that may have influenced some of the results. This could be addressed by including only surveys that were completed in the “average survey completing time-frame.” In addition, it might also be helpful to include “attention check questions” (Van Veldhuisen et al., 2017). Unfortunately, the survey completing time was not recorded and we did not facilitate attention check questions. Both provisions should nevertheless be incorporated in future similar studies.

Cleft-surgeons, orthodontists, and others involved in cleft palate care may take the findings of this study into account during surgical procedures and treatment. This study once again emphasized the importance for cleft-surgeons to strive for a symmetrical nasolabial appearance outcome during primary lip closure and primary nasal correction, avoiding a smaller nostril, and especially a whistling deformity.

Finally, in addition to the 2 objectives outlined in the introduction section: This study could provide input to add additional rules to the CARS. As it was specifically designed to grade “typical” results, raters did not know how to grade the “atypical” results. When the mean rank position of “atypical” photographs in this study is compared to the position of the “typical” photographs that received CARS scores in the study by Mulder et al. (2018), similar CARS scores can be used for the “atypical” outcomes, resulting in a “D” score for atypical nasal outcomes (narrow nostril), a “3” (low vermillion border) and a “4” (whistling deformity) for atypical lip outcomes. However, the main focus should remain on the degree of asymmetry, as the atypical appearance outcome of photograph “Photoshop small” is better than “Small 4” (Table 1). A future study should assess if the suggested scores for these atypical outcomes facilitate the rating process and increase the reliability of the assessment method.

## Conclusion

The findings in this study provide insight into the assessment of “typical” and “atypical” appearance outcomes of the nose and lip after UCLP repair. “Atypical” outcomes, such as noses with a smaller nostril and lips containing a whistling deformity, were perceived as poorer than the more “typical” outcome deformities. Nevertheless, the most symmetrical outcomes were ranked as best, regardless of being a “typical” or “atypical” outcome. Professionals, patients, and laypeople are in agreement when judging these various outcomes on overall appearance.
